# Unilateral Absence of Pulmonary Artery Complicated With Severe Pulmonary Hypertension: Favorable Response to Parenteral Therapy

**DOI:** 10.1016/j.jaccas.2025.104549

**Published:** 2025-08-06

**Authors:** Timothy G. Kuzel, Karim El-Kersh

**Affiliations:** a University of Arizona Internal Medicine Residency Program, Phoenix, Arizona, USA; b University of Arizona College of Medicine, Phoenix, Arizona, USA

**Keywords:** hypoxemia, pulmonary hypertension, right-sided catheterization

## Abstract

**Background:**

Unilateral absence of pulmonary artery (UAPA) is a rare disorder that can present as an isolated lesion or in association with other congenital heart defects. An important complication of UAPA is the development of pulmonary hypertension (PH).

**Case Summary:**

We discuss an adult patient with severe PH in the setting of congenital UAPA and highlight the hemodynamic response to combination therapy that included parenteral treprostinil.

**Discussion:**

To our knowledge, this is the first case report using parenteral treprostinil as part of triple combination therapy to treat PH in setting of UAPA.

**Take-Home Messages:**

There are various treatment modalities for severe PH complicating isolated UAPA. This case highlights an additional option, parenteral treprostinil, for physicians to consider in highly selected cases.

## Visual Summary

**Figure:**
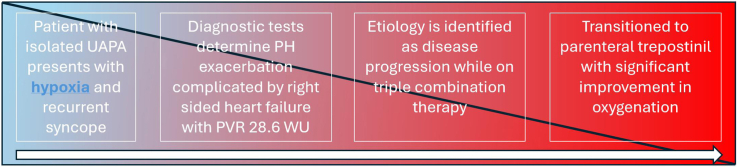

## History of Presentation

A 58-year-old woman presented for evaluation of repeated syncopal episodes on minimal exertion. On presentation, she was found to be severely hypoxemic. The patient's oxygen saturation was 70% on 6 L nasal cannula. Noninvasive supplemental oxygen (high-flow nasal cannula at 50 L/min and fractional inspired oxygen of 0.9) was required to maintain a goal oxygen saturation >90%.

**Take-Home Messages**

• There are various treatment modalities for severe pulmonary hypertension complicating isolated unilateral absence of pulmonary artery.
• This case highlights an additional option, parenteral treprostinil, for physicians to consider in highly selected cases.

## Past Medical History

This patient has a medical history of severe pulmonary hypertension (PH), congenital left unilateral absence of pulmonary artery (UAPA) and left lung hypoplasia, rheumatoid arthritis, and hepatitis C, and a history of cocaine abuse. There was no documented history of cocaine-induced cardiomyopathy. She was NYHA functional class IV and required oxygen at 6 L/min by an Oxymizer at baseline. Two years before presentation, her outside right heart catheterization showed a mean pulmonary artery pressure (mPAP) of 56 mm Hg, a pulmonary artery wedge pressure (PAWP) of 11 mm Hg, a cardiac output (CO) of 3.63 L/min, a cardiac index of 1.9 L/min/m^2^, and a pulmonary vascular resistance (PVR) of 12.4 WU. Findings consistent with cirrhosis and portal hypertension were not observed on abdominal ultrasound. The patient was adherent to her regimen, started by an outside physician, of riociguat 2.5 mg orally 3 times daily, ambrisentan 10 mg orally daily, and selexipag 1,600 mcg orally twice daily.

## Differential Diagnosis

The differential diagnosis for a patient with acute hypoxemia and worsening functional status is broad. This patient has a known pulmonary and cardiac pathology; therefore, our differential focused on the cardiopulmonary systems. Hypoxemia and decreased performance status can be due to decompensated PH, reduced left heart function, reduced right heart function, and pneumonia or pulmonary embolism. The patient's risk factors of UAPA, prior drug use, and lung hypoplasia suggest that she could have components of group 1 and possibly group 3 PH.

## Investigations

**Figure 1:**
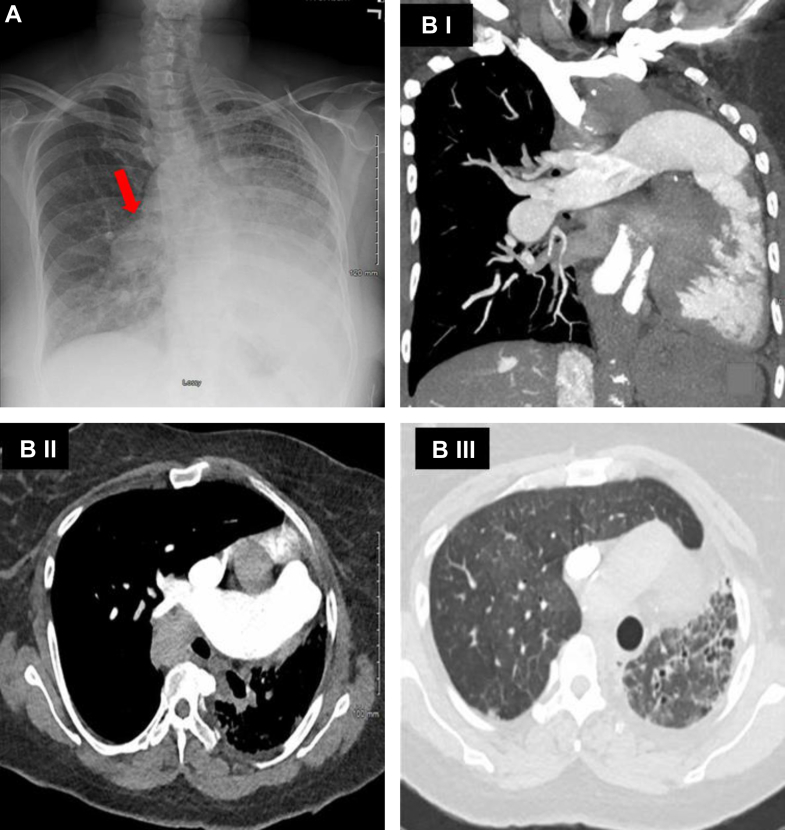
Chest X-Ray and CT Chest With Contrast Chest x-ray (A). The red arrow highlights a right descending pulmonary artery. A finding associated with pulmonary hypertension. Computed tomography chest with contrast (B). Coronal view demonstrating the absence of the left pulmonary artery, enlargement of the main and right pulmonary arteries without pulmonary embolism, hyperinflation of the right lung, and hypoplasia of the left lung (B.I). Transverse view of the chest (B.II). Another transverse view demonstrating hyperinflation of the right lung and hypoplasia of the left lung (B.III).

**Figure 2:**
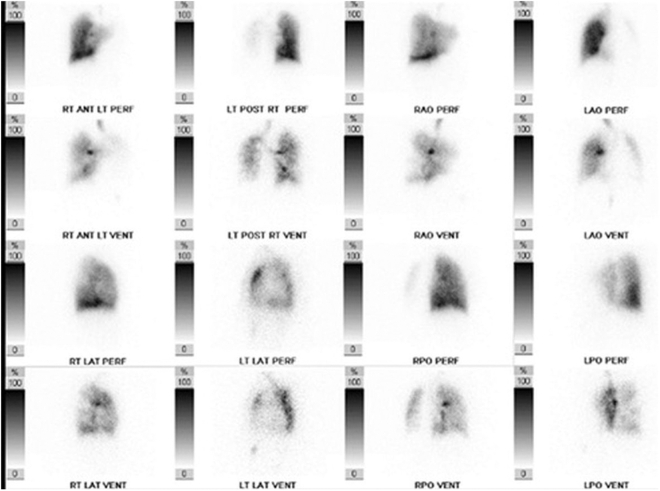
Ventilation-Perfusion Scan Ventilation-perfusion scan demonstrating marked decreased perfusion of the left lung, decreased ventilation of the anterior left lung with some patchy ventilation of the posterior aspect of the left lung, and normal right lung perfusion. ANT = anterior; LAO = left anterior oblique; LAT = lateral; LPO = left posterior oblique; LT = left; PERF = perfusion; POST = posterior; RAO = right anterior oblique; RPO = right posterior oblique; RT = right; VENT = ventilation.

The evaluation revealed an N-terminal pro–B-type natriuretic peptide (NT-proBNP) level of 6,170 pg/mL. The transthoracic echocardiogram showed a left ventricular ejection fraction of 45%, severe right ventricular dilation with severe hypokinesis, and a right ventricular systolic pressure (RVSP) of 100 mm Hg. No atrial or ventricular intracardiac shunts were seen with a contrast echocardiogram. Chest computed tomography with contrast showed the absence of the left pulmonary artery, enlargement of the main and right pulmonary arteries without pulmonary embolism, compensatory enlargement of the right lung, and hypoplasia of the left lung (Figure 1). Subsequent pulmonary function tests showed total lung capacity 64%, forced expiratory volume in 1 second/forced vital capacity 86, forced expiratory volume in 1 second 43%, forced vital capacity 39%, and diffusing capacity of the lungs for carbon monoxide 23% of predicted. A ventilation-perfusion scan showed marked decreased perfusion of the left lung, decreased ventilation of the anterior left lung with some patchy ventilation of the posterior aspect of the left lung, and normal right lung perfusion. Repeat right heart catheterization showed significant worsening of the hemodynamics with an mPAP of 76 mm Hg, a reduced CO of 2.41 L/min, and a PVR of 28.6 WU (Figures 1 and 2, Table 1).

**Table 1:** Hemodynamics Before and After Subcutaneous Treprostinil Initiation

	RHC Before Treprostinil	RHC 2 Months After Starting SQ Treprostinil
mRAP (mm Hg)	11	4
RVP (mm Hg)	106/5	78/1
PAP (mm Hg)	111/55	77/36
mPAP (mm Hg)	76	51
PAWP (mm Hg)	7	7
CO (L/min) (thermodilution)	2.41	4.19
CI (L/min/m^2^) (thermodilution)	1.2	2.2
PVR (WU)	28.6	10.5
RA saturation (%)	40	54
PA saturation (%)	38	53

CI = cardiac index; CO = cardiac output; mPAP = mean pulmonary artery pressure; mRAP = mean right atrial pressure; PA = pulmonary artery; PAP = pulmonary artery pressure; PAWP = pulmonary artery wedge pressure; PVR = pulmonary vascular resistance; RA = right atrium; RHC = right heart catheterization; RVP = right ventricle pressure; SQ = subcutaneous.

## Management

The elevated NT-proBNP with a mild ejection fraction reduction, normal PAWP, but severe right ventricular dilation and an elevated RVSP of 100 mm Hg is suggestive of severe PH. A normal PAWP makes group II PH unlikely. Pulmonary embolism can cause decompensation in someone with underlying PH and compromised pulmonary vascular reserve; however, no pulmonary embolism was seen on chest computed tomography angiography. The right heart catheterization was essential for diagnosis. Elevation of the mPAP and PVR with a normal PAWP is consistent with a precapillary etiology of PH. Patient adherence to her medication regimen was confirmed. The patient was experiencing disease progression despite her current triple PH regimen. Therefore, the patient was transitioned from selexipag to intravenous treprostinil. The goal of treatment was to provide a reduction in oxygen requirement by decreasing her PVR and, in turn, the stress on the right side of the heart. The selexipag dose was simultaneously titrated down while uptitrating treprostinil with continued rapid titration to 40 ng/kg/min over a few days. Diuretics were administered concurrently.

## Outcome

There was improvement in her dyspnea and oxygenation. The patient was weaned from a high-flow nasal cannula to a 6 L/min Oxymizer. The NT-proBNP trended down to 332 pg/mL with no recurrence of syncope. The patient was transitioned from intravenous to subcutaneous treprostinil before discharge. Uptitration of subcutaneous treprostinil continued at home with a target dose of 64 ng/kg/min. After 2 months, a repeat echocardiogram showed improvement with a left ventricular ejection fraction of 67%, moderate right ventricular dilation with mild hypokinesis, and an RVSP of 60 mm Hg. A repeat right heart catheterization showed significant hemodynamic improvement with an mPAP of 51 mm Hg, a CO of 4.19 L/min, and a PVR of 10.5 WU (Table 1).

## Discussion

Unilateral pulmonary artery agenesis is a rare congenital disorder. A total of 187 cases have been reported in the literature from 1868 to 2000.^1^ The agenesis of the right pulmonary artery is more common than the left. UAPA can be associated with cardiac congenital anomalies such as atrial septal defect, tetralogy of Fallot, right aortic arch, coarctation of aorta, and truncus arteriosus but also can occur in isolation such as the case in our patient. Another review that focused on isolated UAPA identified 108 cases between 1978 and 2000, with a median age of 14 years with identification of PH in 44% of the patients.^2^ It is believed that the absence of a pulmonary artery can be related to the involution of the proximal sixth aortic arch with persistence of the connection of the pulmonary artery to the distal sixth aortic arch.^1^ Hemoptysis can complicate these cases as they can develop systemic collateral vessels to the affected lung. Our patient did not report any history of hemoptysis.

Patients with UAPA and lung hypoplasia who develop PH pose a management dilemma as no treatment consensus exists. If discovered during neonatal or infantile years, surgical revascularization of the affected lung is attempted to provide an opportunity for maturation of the affected lung.^3^ If the UAPA is not discovered until later in life, it may manifest with dyspnea on exertion or PH.^4^

Unlike children, UAPA in adults is medically managed.^4^ Prior case reports discuss the use of targeted therapies to treat PH such as endothelin receptor antagonists (ERAs),^5^ ERAs and phosphodiester 5 inhibitors^6^ and riociguat, ERAs, and selexipag.^7^ This is the first case, to our knowledge, to demonstrate utilization of combination therapy including parenteral treprostinil with documented significant hemodynamic improvement after the transition from selexipag. Inhaled treprostinil could be an intriguing option in such cases that are complicated by pulmonary hypoplasia and restrictive physiology because it is the only pulmonary artery vasodilator that is approved for both group 1 and group 3 PH-interstitial lung disease and has the theoretical benefit of avoiding worsening ventilation perfusion mismatch. Given the severity of our patient's presentation and significant disease progression that occurred while on triple therapy, we chose to switch to parenteral therapy. Despite her restrictive physiology due to the associated pulmonary hypoplasia, significant improvement in hemodynamics and oxygenation was noted. Our patient met several clinical, echocardiographic, and hemodynamic high-risk parameters with her severe PH. Although mainly used for group 1 stratification, there is growing evidence that risk assessment could be of value for mixed PH groups as well.^8^, ^9^, ^10^

## Conclusions

Isolated UAPA is a rare congenital disorder that can be complicated by PH. Careful phenotyping of these patients by PH experts is important to decide the best management strategy under close observation. Vascular phenotype with significant precapillary PH could benefit from pulmonary artery vasodilator therapies in different combinations.

**Visual Summary:**
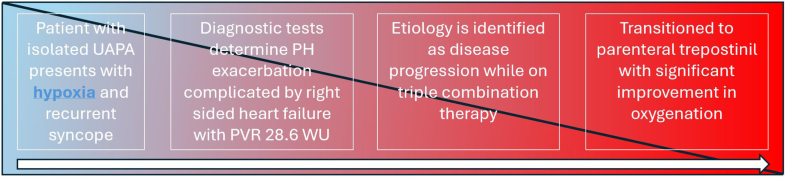
Sequence of Events PH = pulmonary hypertension; PVR = pulmonary vascular resistance; UAPA = unilateral absence of pulmonary artery.

## Funding Support and Author Disclosures

Dr El-Kersh serve as consultants for Merck, J&J, and United Therapeutics. All other author has reported that they have no relationships relevant to the contents of this paper to disclose.
